# Competing endogenous RNA network analysis identifies critical genes among the different breast cancer subtypes

**DOI:** 10.18632/oncotarget.14361

**Published:** 2016-12-29

**Authors:** Juan Chen, Juan Xu, Yongsheng Li, Jinwen Zhang, Hong Chen, Jianping Lu, Zishan Wang, Xueying Zhao, Kang Xu, Yixue Li, Xia Li, Yan Zhang

**Affiliations:** ^1^ College of Bioinformatics Science and Technology, Harbin Medical University, Harbin, China

**Keywords:** breast cancer, subtypes, miRNA, competing endogenous RNAs, network hubs

## Abstract

Although competing endogenous RNAs (ceRNAs) have been implicated in many solid tumors, their roles in breast cancer subtypes are not well understood. We therefore generated a ceRNA network for each subtype based on the significance of both, positive co-expression and the shared miRNAs, in the corresponding subtype miRNA dys-regulatory network, which was constructed based on negative regulations between differentially expressed miRNAs and targets. All four subtype ceRNA networks exhibited scale-free architecture and showed that the common ceRNAs were at the core of the networks. Furthermore, the common ceRNA hubs had greater connectivity than the subtype-specific hubs. Functional analysis of the common subtype ceRNA hubs highlighted factors involved in proliferation, MAPK signaling pathways and tube morphogenesis. Subtype-specific ceRNA hubs highlighted unique subtype-specific pathways, like the estrogen response and inflammatory pathways in the luminal subtypes or the factors involved in the coagulation process that participates in the basal-like subtype. Ultimately, we identified 29 critical subtype-specific ceRNA hubs that characterized the different breast cancer subtypes. Our study thus provides new insight into the common and specific subtype ceRNA interactions that define the different categories of breast cancer and enhances our understanding of the pathology underlying the different breast cancer subtypes, which can have prognostic and therapeutic implications in the future.

## INTRODUCTION

Non-coding RNAs are a class of RNAs that regulate gene expression transcriptionally and post-transcriptionally [1]. The miRNAs are a class of ∼22-nucleotide long single-stranded non-coding RNAs that regulate gene expression by binding to MREs (miRNA response elements) on the RNAs [2]. Aberrant miRNA expression patterns have been associated with many human cancers including breast cancer [3]. The lncRNAs (long non-coding RNAs) are another class of non-coding RNAs (longer than 200 nucleotides), that are also involved in the pathology of many complex human diseases including cancers [4]. The lncRNAs also harbor MREs and compete with other RNAs for miRNA binding, thus can function as competing endogenous RNAs (ceRNAs) to influence the post-transcriptional regulation in cancers [5, 6].

Breast cancer is a progressive disease that is a leading cause of cancer-related mortality in women and composed of distinct subtypes that are highly heterogeneous [7–10]. Based on gene expression, breast cancer is classified into five major subtypes namely luminal A, luminal B, HER2-enriched, basal-like, and normal-like [11, 12]. The luminal subtypes (A and B) are characterized by the expression of ER (estrogen receptor) and/or PR (progesterone receptor) related genes [13]. The HER2-enriched subtype is often associated with frequent HER2/ERBB2 amplification (80%) and therefore HER2/ERBB2 is targeted for therapy [14]. The basal-like subtype is generally negative for ER, PR and HER2 and therefore also called as triple-negative breast cancer or TNBC, that is characterized by enhanced invasiveness and poor clinical outcomes [15]. The etiology of different breast cancer subtypes shows variant risk factors that may result in different clinical outcomes [16, 17]. Therefore, understanding the similarities and differences among the various subtypes is important from the clinical standpoint.

Although many breast cancer biomarkers have been reported [18, 19], it has come to light that many of these biomarkers are coordinately regulated by similar miRNAs that implies competition among shared miRNAs to bind to different RNAs. For example, Li et al. found that the STARD13-correlated ceRNA network inhibits epithelial-mesenchymal transition (EMT) and metastasis of breast cancer [20]. However, these studies focused on the clinical utility of individual ceRNA interactions and did not investigate the ceRNA interactions at a system level. Xu et al. conducted a systemic analysis of ceRNA interactions in breast cancer without considering the heterogeneity of breast cancer subtypes and the involvement of lncRNAs [21]. Also, studies investigating ceRNA interactions in human cancer have focused on dysregulated RNAs that are aberrantly expressed during cancer initiation and progression [22]. Due to the heterogeneity of breast cancers, the expression profiling is more complex [7, 23]. Hence, a systemic analysis of ceRNA crosstalk among different breast cancer subtypes may yield better information regarding the interplay of various biological networks that are involved in breast carcinogenesis [24]. Furthermore, since the potential interactions between the subtype molecular biomarkers have not been considered in previous studies, we opined that the ceRNA network analysis would help dissect the pathway interactions at a molecular level.

Therefore, the aim of this study was to generate subtype specific dysregulated miRNA mediated ceRNA networks to discover new critical subtype related genes and to unravel the shared and specific pathogenic pathways of the breast cancer subtypes at a system level

## RESULTS

### The miRNA dys-regulatory networks in the breast cancer subtypes

**Figure 1:**
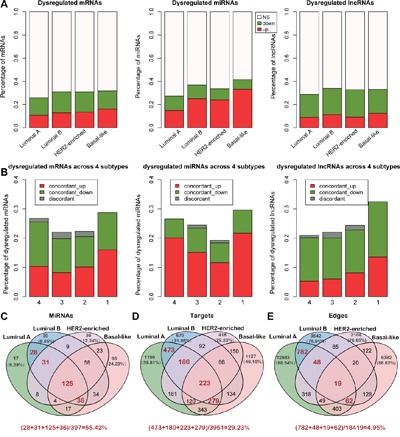
Dysregulated coding and non-coding RNAs among different breast cancer subtypes **A.** The percentage of dysregulated mRNAs, miRNAs and lncRNAs across four breast cancer subtypes (Wilcoxon Rank Sum tests, FDR<0.05). Note: ‘NS’ means no significant difference in expression between the breast cancer subtypes and normal samples. **B.** The percentage of concordant and discordant changes of RNAs that are dysregulated in 4, 3, 2 and specific subtypes, respectively. Concordant change is defined as the expression of a RNA changed in the same direction across all breast cancer subtypes. **C-E.** The Venn diagram depicting the nodes, target nodes and edges of the miRNAs across the four breast cancer miRNA dys-regulatory subtype networks. The red digit indicates the number of shared nodes or edges between the luminal A and the luminal B miRNA dys-regulatory networks. The percentages of shared nodes (or edges) between the luminal A and the luminal B subtype networks were calculated and showed under each Venn diagram.

To systematically identify disease markers in each of the four breast cancer subtypes, we performed Wilcoxon Rank Sum tests to identify differentially expressed protein coding and non-coding genes in each of the breast cancer subtypes (Figure 1A, Supplementary Table 1). Each subtype exhibited about 40% differentially expressed coding and non-coding genes (Figure 1A). Further, majority of the miRNAs were upregulated and most of the lncRNAs were downregulated in all the subtypes. About 70% of dys-regulated coding and non-coding RNAs are shared in at least two breast cancer subtypes (Figure 1B). Also, about 95% of subtype shared dysregulated mRNAs, miRNAs and lncRNAs are concordantly changed across breast cancer subtypes, comparing the four breast cancer subtypes, namely luminal A, luminal B, HER2-enriched and basal-like (Figure 1B). These data indicated that these shared dys-regulated RNAs may result in accordant direction change of cancer-related pathways in different breast cancer subtypes. Among the subtypes, basal-like exhibited the highest fraction of dysregulated coding and non-coding RNAs that lead to malignancy as shown in supplementary Table 2 [15, 25]. Notably, several subtype marker genes were identified that were consistent with previous studies like ESR1, PGR (progesterone receptor) and FOXC1 [11]. For example, ER, ESR1 and PGR genes were downregulated in the basal-like subtype, but, upregulated in the luminal A/B subtypes. Similarly, FOXC1 was upregulated in the basal-like subtype and down-regulated in the luminal A/B and HER2-enriched subtypes.

Regarding miRNAs, previous studies have suggested that miRNAs usually function as negative regulators of gene expression [2, 26]. Therefore, we constructed the subtype miRNA dys-regulatory networks by assuming that the miRNAs negatively regulated their target genes (Supplementary Table 3). Our data suggested that most of the miRNA targets were subtype specific. However, the subtypes with similar tissue-of-origin, such as the luminal A and the luminal B shared many nodes and edges in the networks (Figure 1C-1E). Also, the network structure of the miRNA dys-regulatory networks was organized according to a core set of principles and not randomly. This suggested that their analysis would help understand the regulation between the miRNAs and their targets at a system level (Supplementary Figure 1A-1B).

### CeRNA networks among the breast cancer subtypes

**Figure 2:**
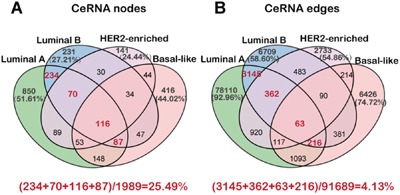
The nodes and edges of ceRNA networks across four breast cancer subtypes **A-B.** The Venn diagram showing the nodes and edges across the four breast cancer subtype ceRNA networks.

To investigate the ceRNA regulation in breast cancer subtypes, we generated a ceRNA network for each subtype by assembling all the significant ceRNA interactions that were based on the significance of both, their positive co-expression and the shared miRNAs, in the corresponding subtype miRNA dys-regulatory network (Table 1). Accordingly, we identified 84,026, 11,449, 4,982 and 8,600 ceRNA interactions in the networks of luminal A, luminal B, HER2-enriched and basal-like subtype, respectively. We found 26.88%, 11.79%, 8.07% and 12.54% of dysregulated mRNAs as well as 6.50%, 2.58%, 1.75% and 3.30% of dysregulated lncRNAs in the ceRNA networks of luminal A, luminal B, HER2-enriched and basal-like subtype, respectively. This indicated that more dys-regulated mRNAs are influenced by the ceRNA regulation mechanism compared with lncRNAs. Further, we observed that many ceRNAs were well connected and formed a tight network in each subtype (Supplementary Figure 2A-2D). Similar to the miRNA dys-regulatory networks, the ceRNA networks showed that the ceRNA interactions are much more specific to each subtype in spite of overlapping nodes among the four different subtypes, suggesting unique subtype specific regulation (Figure 2A-2B). As expected, the luminal A and B subtypes that are similar in the tissue of origin and at the molecular level showed greater overlap of the ceRNA network (Figure 2A-2B). We also observed that the degree distribution of the ceRNA networks satisfied the power law model suggesting that the networks were scale-free and not randomly organized implying rapid communication among different ceRNAs (Supplementary Figure 2E).

**Table 1:** The number of nodes and edges which are involved in ceRNA networks across four breast cancer subtypes

	Edges	Nodes (lncRNA)	Nodes (mRNA)	Nodes(miRNA)
Luminal A	84,026	237	1,410	164
Luminal B	11,449	111	738	154
HER2-enriched	4,982	73	504	101
Basal-like	8,600	139	806	184

Several marker genes of specific breast cancer subtypes have been reported to be differentially expressed [9, 11]. We observed that these marker genes were highly connected in the ceRNA networks. For example, in luminal A breast cancer, EGFR and FOXC1 had 453 and 343 ceRNA connections, respectively and shared four miRNAs (miR-15b-3p, miR-141-3p, miR-200a-3p and miR-760). This suggested that the marker genes of breast cancer subtypes can co-regulate each other and have high connectivity in the ceRNA networks.

### Subtype common ceRNAs tend to be at the core of ceRNA networks

**Figure 3:**
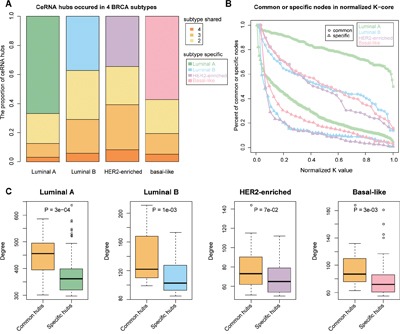
Common subtype ceRNAs are at the core in the ceRNA networks **A.** The percentage of hub ceRNAs distributed in 1–4 breast cancer subtypes **B.** Relationships between ceRNA layers and the percentage of specific or common subtype ceRNAs in each layer. Increasing layer numbers correspond to regions of increasing densities in the network. The layers of each subtype network were normalized to 0-1 and the frequencies were accumulated. **C.** The difference in degrees between common and specific subtype ceRNA hubs (Wilcoxon Rank Sum test). Yellow colored boxes represent the degree distribution of common subtype ceRNAs and other colored boxes correspond to subtype specific ceRNA hubs.

A key network property is node connectivity that shows the degree of communication among the nodes. Hub node is pivotal and shows high connectivity with other nodes. In our study, we defined the hubs as the top 10% of the nodes with the highest degree within the networks. The hub nodes were sub-divided into subtype common and subtype specific ceRNA nodes that would identify mechanisms that are either shared or specific among the different breast cancer subtypes. The subtype common ceRNA hubs were defined as ceRNA hubs that occurred in at least three of the subtype ceRNA networks and accounted for 12.65%, 29.07%, 39.34% and 19.39% of total ceRNA hubs in luminal A, luminal B, HER2-enriched and basal-like, respectively (Figure 3A). On the other hand, the subtype specific ceRNA hubs were defined as hubs that occurred in each of the specific subtype networks only. Also, the luminal A and basal-like had high proportion of subtype specific ceRNA hubs relative to common ceRNA hubs.

While the node degree of a network represents the local centrality, k-core decomposition reveals the modularity of central hub nodes and is used to identify the largest sub-graph where the vertices have at least ‘k’ connections within a subgraph called as the k-cores [27]. Therefore, hubs that are closely connected with other hub nodes are within k-cores have larger k values, whereas, hubs closely connected to non-hub nodes have smaller k values. When we conducted k-core decomposition analysis to analyze the localization of common and subtype specific nodes, we observed that the subtype common ceRNAs had larger k values compared to subtype-specific ceRNA nodes (Figure 3B). This indicated greater centrality in the networks and suggested that all breast cancer subtypes shared pathogenic pathways. Also, the Wilcoxon Rank Sum test revealed that the subtype common ceRNA hubs had higher connectivity compared with subtype-specific ceRNA hubs in each network, which may provide some clues of common biological processes disturbed among the different breast cancer subtypes (Figure 3C).

### Subtype common ceRNA hubs are associated with proliferation in breast cancers

**Figure 4:**
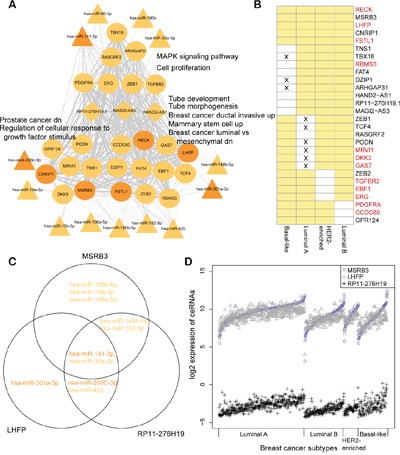
Common subtype ceRNA hubs are associated with breast cancer proliferation **A.** Common subtype ceRNA hub sub-network. The solid line shows ceRNA interactions and the dotted line shows target miRNA relationships. The width of an edge is based on the number of subtype ceRNA networks in which the ceRNA interaction occurred. Only common hub miRNAs were considered in this network. Some significantly enriched gene sets are listed alongside. Up/dn means upregulation/downregulation in the corresponding gene sets of msigDB. **B.** Common ceRNA hubs were defined as hubs in different subtype ceRNA networks. Yellow box refers to the ceRNA which is defined as a hub in the corresponding subtype ceRNA network. The cross denotes that the expression of a ceRNA has no significant change in the corresponding subtype compared with normal breast tissues. Otherwise it is considered dysregulated. The red ceRNAs indicate cancer genes listed in CGC or GAD. **C.** An example of miRNA regulations for ceRNA interactions among MSRB3, LHFP and RP11-276H19(ENSG00000226237). Only the common hub miRNAs were considered and the colors are concordant with the networks in figures S2A-S2D. **D.** The expression of ceRNAs in different subtypes are shown (log2 transformed). The clinical samples were sorted by the expression of MSRB3 for each breast cancer subtype.

Next, we analyzed the relationship among common ceRNA hubs to understand their regulation of breast cancer progression. Towards this, we collected all the ceRNA interactions among the 28 common ceRNA hubs in the four breast cancer subtypes to form a subtype common sub-network (Figure 4A-4B). Five of the twenty eight ceRNA hubs were common to all the four breast cancer subtype networks, including RECK, which controls breast cancer metastasis by regulating MMP-2 (matrix metalloproteinases-2) to inhibit tumor angiogenesis [28]. We also found that these ceRNA hubs were highly connected with each other and regulated by subtype common hub miRNAs in the corresponding miRNA dys-regulatory network (Figure 4A). Furtherly, 147 of the 353 ceRNA interactions among the 28 subtype common ceRNA hubs occurred in at least three breast cancer subtypes. The subtype common ceRNA network was regulated by 13 common hub miRNAs in at least three miRNA dys-regulatory networks, including breast cancer related miR-141-3p, miR-33a-5p, miR-200c-3p, and miR-182-5p [29–31]. We also identified 25 protein coding genes among the 28 subtype common ceRNA hubs, of which 12 (RECK, LHFP, FSTL1, TGFBR2, EBF1, ERG, MRVI1, DKK3, GAS7, RBMS3, PDGFRA and CCDC80) were listed as cancer related genes in the Cancer Gene Census (CGC) of the COSMIC database or the Genetic Association Database (GAD) [32, 33]. LHFP, a common ceRNA hub in all the four breast cancer subtypes is associated with mesenchymal differentiation in glioma [34]. Defective MSRB3 has been shown to inhibit cell proliferation through the activation of p53-p21 and p27 pathways [35]. Also, MSRB3 and LHFP co-regulate each other by sharing common hub miRNAs, miR-141-3p and miR-33a-5p (Figure 4C-4D; Pearson correlation coefficients are 0.74, 0.74, 0.65 and 0.63 in luminal A, luminal B, HER2-enriched and basal-like, respectively).

Furthermore, our data showed that the lncRNAs functioned as ceRNAs involved in the cancer related biological processes. We found RP11-276H19.1 (ENSG00000226237) as a common hub that participated in the ceRNA interaction networks in luminal A, luminal B and HER2-enriched subtypes. It also co-regulated with MSRB3 and LHFP by sharing miRNAs to form ceRNA-ceRNA interactions in the three subtypes (Figure 4C-4D). Also, the expression of these three ceRNAs had similar trend in each breast cancer subtype (Figure 4D). Functional enrichment analysis of the ceRNA hubs revealed their involvement in proliferation (FDR = 4.80e-03, hypergeometric test, Bonferroni corrected), MAPK signaling pathway (FDR = 4.10e-03), tube morphogenesis (FDR = 4.37e-03) and some breast cancer associated processes (Table 2, Supplementary Table 4), demonstrating that the proliferation process was altered substantially in all breast cancer subtypes [36].

**Table 2:** Enriched gene sets for common ceRNA hub subnetwork

Category of Gene sets	Gene set name	Count	FDR
	tube morphogenesis	5	4.37e-03
	regulation of cellular response to growth factor stimulus	4	4.80e-03
**GO_BP**	regulation of transforming growth factor beta receptor signalling pathway	3	4.80e-03
	tube development	5	4.83e-03
**KEGG**	MAPK signalling pathway	3	4.10e-03
	schuetz breast cancer ductal invasive up	9	5.43e-09
**CGP**	charafe breast cancer luminal vs mesenchymal dn	10	5.43e-09
	lim mammary stem cell up	10	5.43e-09
	liu prostate cancer dn	7	1.65e-05

Note: BP is the abbreviation of ‘Biological Process terms for Gene Ontology’ ; KEGG is the abbreviation of ‘Kyoto Encyclopedia of Genes and Genomes’; CGP is the abbreviation of ‘chemical and genetic perturbations’.

### Subtype specific ceRNA hubs contribute to breast cancer subtype phenotype

**Figure 5:**
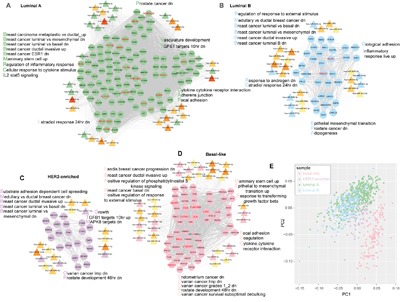
Subtype specific ceRNA hubs contribute to breast cancer subtype phenotype **A-D.** Subtype specific ceRNA hub sub-networks are shown. The solid line depicts ceRNA interactions and the dotted line shows target miRNA relationships. The hubs with red gene label correspond to the 29 critical subtype-specific ceRNA hubs. Only hub miRNAs were considered in each network. Some of the significantly enriched gene sets are listed alongside. Up/dn means upregulation/downregulation in the corresponding gene sets of msigDB. **E.** Principal component analysis (PCA) plots showing distinct populations identified in the four breast cancer subtypes (based on the ggfortify R package). ‘PC’ stands for principal component.

To discover the pathogenic mechanisms of different breast cancer subtypes, we further focused on the subtype specific ceRNA hubs. We extracted four subtype-specific ceRNA hub sub-networks by collecting all subtype-specific ceRNA hubs and their ceRNA interactions within each of the four subtype ceRNA networks and found that the subtype-specific hubs were highly connected with each other and regulated by hub miRNAs in the miRNA dys-regulatory network of each subtype (Figure 5A-5D; Table 3; Supplementary Table 5). Several miRNA hubs were subtype specific and had specific roles in the corresponding breast cancer subtype. For instance, the three members of the miR-17-92 cluster (miR-17-3p, miR-20a-5p and miR-19b-3p) were part of an oncogenic cluster and associated with lymph node metastases in TNBC [37]. These three miRNAs were significantly upregulated in basal-like subtype compared with normal breast tissue. In addition, KDR or VEGF2, is also a basal-like subtype specific hub and a specific biomarker in TNBC based on a long-term follow-up dataset [38]. KDR acted as a ceRNA partner of PGR, another basal-like specific hub and competing for the binding to the miR-17-92 cluster (including miR-17-3p, miR-19b-1-5p and miR-20a-5p). Interestingly, we found that these subtype-specific ceRNA hubs participated in breast or other hormone related cancer like prostate cancer, ovarian cancer and endometrial cancer (Table 3, Supplementary Table 5), consistent with previous findings that hormone related cancers had similar pathways [39]. In addition, some biological pathways were identified to be assigned to specific subtypes. For example, estradiol response and IL2 -STAT5 signaling (inflammatory response pathway) were luminal A and luminal B specific (Table 3), whereas, the coagulation pathway was specifically involved in the basal-like subtype (FDR = 1.16e-02).

**Table 3:** Enriched gene sets for subtype specific ceRNA hub subnetworks

Subtypes	Category	Gene set name	Count	FDR
	HALLMARK	IL2 stat5 signalling	4	4.48e-02
	GO_BP	regulation of inflammatory response	6	2.23e-02
		cellular response to cytokine stimulus	9	2.23e-02
	KEGG	focal adhesion	6	1.01e-02
		adherens junction	3	2.53e-03
		cytokine cytokine receptor interaction	5	36.2e-02
LuminalA		charafe breast cancer luminal vs mesenchymal dn	23	3.47e-14
		liu prostate cancer dn	23	5.04e-14
		lim mammary stem cell up	23	8.74e-14
	CGP	charafe breast cancer luminal vs basal dn	15	5.14e-07
		doane breast cancer ESR1 dn	4	1.14e-03
		dutertre estradiol response 24hr dn	9	7.04e-03
		lien breast carcinoma metaplastic vs ductal up	3	2.72e-02
	GO_BP	adipogenesis	3	3.83e-03
		biological adhesion	8	4.20e-03
		regulation of response to external stimulus	7	9.12e-03
	KEGG	vascular smooth muscle contraction	4	2.95e-05
LuminalB	CGP	schuetz breast cancer ductal invasive up	11	3.07e-10
		liu prostate cancer dn	11	3.77e-09
		charafe breast cancer luminal vs mesenchymal dn	8	8.31e-06
		smid breast cancer luminal b dn	7	1.53e-04
		charafe breast cancer luminal vs basal dn	6	3.67-04
		dutertre estradiol response 24hr dn	5	3.07e-03
		doane response to androgen dn	3	1.10e-02
	HALLMARK	epithelial mesenchymal transition	6	1.57e-06
		substrate adhesion dependent cell spreading	3	4.39e-04
	GO_BP	growth	4	7.58e-03
		biological adhesion	6	7.58e-03
	KEGG	focal adhesion	3	1.08e-03
		schuetz breast cancer ductal invasive up	10	3.85e-11
**HER2-enriched**		liu prostate cancer dn	6	1.30e-04
		wamunyokoli ovarian cancer lmp dn	3	2.21e-03
	CGP	charafe breast cancer luminal vs mesenchymal dn	4	2.32e-03
		plasari TGFB1 targets 10hr up	3	2.32e-03
		yoshimura MAPK8 targets dn	3	8.44e-03
		schaeffer prostate development 48hr dn	3	1.20e-02
		charafe breast cancer luminal vs basal dn	3	1.26e-02
	HALLMARK	epithelial mesenchymal transition	7	5.49e-06
		coagulation	3	1.16e-02
	GO_BP	response to transforming growth factor beta	4	1.90e-02
	KEGG	positive regulation of phosphatidylinositol 3 kinase signaling	3	1.90e-02
		cytokine cytokine receptor interaction	4	1.15e-02
		focal adhesion	3	1.67e-02
**Basal-like**		wamunyokoli ovarian cancer lmp dn	9	2.11e-07
		wong endometrium cancer dn	7	2.10e-07
		schuetz breast cancer ductal invasive up	10	1.41e-06
		smid breast cancer basal dn	12	7.27e-06
	CGP	wamunyokoli ovarian cancer grades 1 2 dn	5	1.83e-05
		bonome ovarian cancer survival suboptimal debulking	8	5.30e-04
		landis breast cancer progression dn	3	4.06e-03
		schaeffer prostate development 48hr dn	6	4.09e-03

Note: GO_BP is the abbreviation of ‘Biological Process terms for Gene Ontology’; KEGG is the abbreviation of ‘Kyoto Encyclopedia of Genes and Genomes’; CGP is the abbreviation of ‘chemical and genetic perturbations’.

Based on these data, we hypothesized that the subtype-specific ceRNA hubs that took part in subtype specific pathways could be used to classify different breast cancer subtypes. We also performed the principal component analysis (ggfortify R package) and found that the 29 subtype-specific ceRNA hubs that were dysregulated among all breast cancer subtypes (Wilcoxon Rank Sum test, FDR<0.05) could distinguish the basal-like, HER2-enriched and luminal subtypes, whereas distinguishing the luminal A and B subtypes was not clear-cut (Figure 5E).

Taken together, these results suggested that investigating the properties of ceRNA interaction networks may help strengthen our understanding of different subtype breast cancers, which may contribute to breast cancer subtype phenotype, thus further to improve clinical prevention and treatment.

## DISCUSSION

In this study, we developed a computational framework to construct and analyze ceRNA networks using the transcriptional profiling data to understand the dynamic clinical behaviors and outcomes of different breast cancer subtypes.

In this study, we adopted a three-step design to construct the ceRNA interaction network in each subtype to eliminate false discoveries [40, 41]. Thereafter, we used highly stringent threshold values to construct miRNA dys-regulatory networks and the identified common and subtype-specific ceRNA hubs were not influenced by different threshold effects (Supplementary Table 6). In addition, the maximal FDRs for luminal A, luminal B, HER2-enriched and basal-like subtypes were 0, 2.06e^-12^, 1.18e^-04^, 7.11e^-09^, respectively, suggesting that the ceRNA interactions were reliable. We further validated the accuracy of predicting the ceRNA interactions globally by analyzing expression datasets for the four breast cancer subtypes from another array, GSE45827, and found that the ceRNA interaction pairs showed higher positive co-expression compared with the gene pairs that were randomly selected (Supplementary Figure 3).

Further, although majority of the ceRNA nodes and interactions were subtype specific, several common ceRNAs were also shared by the four breast cancer subtypes (Figure 2A and 2B). These common ceRNA hubs indicate shared dynamic changes in many biological processes like MAPK signaling pathway, proliferation process and tube morphogenesis during breast tumorigenesis (Table 2). Conversely, the subtype specific ceRNA hubs provide clues to specific changes during the transformation of the normal cells into the different breast cancer subtypes (Table 3). For example, our analysis showed that luminal A and luminal B were associated with estradiol response and inflammatory response pathway. This validated our analysis since the luminal subtypes are recognized as estrogen receptor positive breast cancer (ER+) and are clinically treated with hormone therapy [13]. Also, interleukin-6 that is secreted by the transformed fibroblasts induces tamoxifen resistance in luminal breast cancer [42]. On the other hand, we found that the hallmark of the basal-like breast cancer subtype was coagulation pathway, consistent with previous reports that basal breast cancer cells constitutively express coagulation initiator, an alternatively spliced tissue factor (asTF) that promotes breast cancer growth in a β1 integrin-dependent manner [43].

In regard to the tumor suppressive and the oncogenes reported [42], our study demonstrated that both the common and the subtype-specific ceRNA hubs were enriched in tumor suppressive genes and depleted in oncogenes (Supplementary Table 7). To explain this, it has to be noted that the ceRNA hubs are down-regulated in each of the breast cancer subtypes. Therefore, the dysfunction of the enriched tumor suppressor genes probably drives the initiation and progression of breast cancer. Several cancer associated lncRNAs including the common ceRNA hubs like HAND2-AS1 (DEIN) [44, 45] and RP11-276H19.1 [46], as well as subtype-specific hubs like ADAMTS9-AS2, a luminal A specific hub lncRNA, acted as tumor suppressors and inhibited the migration of the glioma cells [47]. Similarly, functional analysis of the miRNA hubs by the TAM tool showed that the common and specific miRNA hubs play important roles in breast cancer subtypes (Supplementary Table 8) [48].

Finally, our analysis revealed 29 critical ceRNA hub genes that could distinguish the basal-like, HER2-enriched and luminal breast cancer subtypes. The Luminal A/B subtypes were similar as they shared many expression markers within the luminal epithelial cell layers. HER2-enriched and luminal A/B subtypes could not be well distinguished as several HER2-enriched subtype showed the expression of ER or PR as well as a global gene expression profile that was similar to the luminal A and B subtypes [49, 50]. Among the 29 subtype related genes, four were part of the 50-gene PAM50 model (FOXC1, SFRP1, PGR and EGFR) [12]. Although the other 25 genes were not part of the PAM50 model, they probably compete for miRNA binding and thus influence the subtype specificities. For example, DCN (decorin) regulates PGR by competing for miR-19b-1-5p and promotes the basal-like subtype (Pearson correlation coefficient =0.54). This supports previous findings that DCN is down-regulated and influences the breast cancer cell motility and invasion, and acts as one of the six-gene signature that adds prognostic value independent of the expression of ER, PR, and HER2 [51, 52]. Therefore, subtype specific expression of 50-gene in PAM50 model can be regulated by other ceRNAs that may influence breast cancer subtype specificity. Interestingly, three of the 29 subtype-specific ceRNA hubs, namely EGFR, IL6ST and MET, are listed in the Cancer Gene Census of the COSMIC database [32]. Therefore, all these data put together identify these 29 genes as crucial for breast cancer subtype determination, maintenance and function and need to be further analyzed in detail.

Systematic studies have shown that somatic mutations are the driving force for human carcinogenesis [53]. In our study, among the 28 common ceRNA hubs, we found 18 mutant genes in at least one breast cancer subtype. Therefore, when we analyzed gene mutations in the general ceRNA networks, we observed that the mutated ceRNAs are highly connected than the other ceRNAs within the four ceRNA networks (Supplementary Figure 4A, Wilcoxon Rank Rum test). This also showed that mutated ceRNAs were enriched in the hubs across all the four breast cancer subtypes (Supplementary Figure 4B, hypergeometric test). Moreover, k-core sub-network analysis indicated that the mutated ceRNAs were localized in the highly dense layers of each ceRNA network, especially in the HER2-enriched and the basal-like subtypes (Supplementary Figure 4C). Although the effects of somatic mutation in human cells is largely unknown [54], since mutated genes had greater ceRNA interactions, we postulate that somatic mutation may drive breast cancer initiation and progression by dysregulating the normal ceRNA interactions.

Therefore, in conclusion, our study provides a framework to compare disease subtypes based on miRNA mediated ceRNA interactions. The systemic network analyses provide understanding of the biological mechanisms underlying the different breast cancer subtypes and help identify new critical genes for each of the breast cancer subtypes. We predict that systematic characterization of ceRNA-ceRNA interactions will contribute to improving the subtype classification and also identifying novel prognostic markers that would help selecting appropriate subtype specific therapies that would improve the survival outcomes for breast cancer patients.

## MATERIALS AND METHODS

### Genome wide transcriptome and mutation datasets of the five breast cancer subtypes

The mRNA and miRNA expression datasets measured by RNA sequencing were downloaded from The Cancer Genome Atlas (TCGA) [23], whereas, the lncRNA sequencing datasets were obtained from The Atlas of ncRNA in Cancer (TANRIC) [55]. The molecular subtype information of breast cancer was downloaded from Synapse (https://www.synapse.org/, syn1461151) for the luminal A, luminal B, HER2-enriched, basal-like and normal-like, with normal breast samples [23]. As the normal-like subtype has high percentage of normal sample “contamination” in the tumor specimen [12], it was excluded from further analysis (Supplementary Table 1). The mutation datasets of each tumor sample were also obtained from Synapse. To filter out lowly expressed RNA transcripts (mRNA, miRNA, lncRNA), the one with expression value of 0 in all of the samples was removed. Finally, 20,211 mRNAs, 12,697 lncRNAs and 1,026 miRNAs were considered for further analysis. All the expression profiles were log2 transformed.

### The miRNA-target relationships

To obtain the regulatory relationships between miRNAs and mRNAs (or lncRNA), miRanda algorithm was used to predict the miRNA-target relationships. As a result, 1,129,584 unique miRNA-lncRNA and 2,927,669 unique miRNA-mRNA relationships were generated among 1,017 miRNAs, 7,731 lncRNAs and 17,215 mRNAs, respectively.

### Functional annotation datasets

Functional annotation gene sets were downloaded from the Molecular Signatures Database (MsigDB) [56]. This included 50 hallmark gene sets, 825 Biological Process (BP) terms for Gene Ontology (GO), 3396 chemical and genetic perturbations (CGP) and 186 Kyoto Encyclopedia of Genes and Genomes (KEGG) gene sets.

### Overview of the construction of subtype ceRNA networks

A three-step model was proposed to construct ceRNA networks. Firstly, we identified differentially expressed mRNA, miRNAs and lncRNAs for each of the breast cancer subtypes compared with normal breast samples. Secondly, four miRNA dys-regulatory networks were constructed by considering negative regulation between dysregulated miRNAs and their targets. Thirdly, based on the miRNA dys-regulatory networks, ceRNA interactions were identified by considering significant sharing of the the regulatory miRNAs and their co-expression. All ceRNA interactions were assembled into a ceRNA network for each breast cancer subtype, where the nodes were referred to as ceRNAs and the edges referred to as ceRNA interactions.

### Identification of differentially expressed mRNAs, miRNAs and lncRNAs

Wilcoxon Rank Sum tests were used to identify differentially expressed protein coding genes and non-coding genes between each subtype and normal breast samples. We applied the Bonferroni correction and the RNAs that showed a FDR less than 0.05 and the absolute log2 fold change (LFC) no less than 1 were considered significant.

### Construction of subtype miRNA dys-regulatory networks

Differentially expressed target genes (mRNAs and lncRNAs) for each differentially expressed miRNA were collected and a miRNA dys-regulatory network was constructed for each subtype by considering negative regulation between miRNAs and their targets based on the expression profiles of each breast cancer subtype samples (Pearson Correlation Coefficient<0, P<0.01).

### Construction of subtype ceRNA networks

The subtype ceRNA interaction networks were constructed based on the miRNA dys-regulatory networks. Two target genes were considered to be competing only if 1) they significantly shared regulatory miRNAs in the miRNA dys-regulatory network as calculated by the hypergeometric test (FDR<0.01, Bonferroni corrected) and 2) the expression of ceRNA-ceRNA interactions showed a statistically significant positive correlation with a Pearson correlation coefficient that was no less than 0.5 (FDR<0.01, Bonferroni corrected). All the ceRNA interactions were assembled into a ceRNA network for each breast cancer subtype and the weights of all edges were set to 1.

### K-core decomposition analysis of the ceRNA networks

The k-core is the largest sub-graph where every node has at least k links. The k-core can be determined by pruning all the vertices from a network with degrees less than k and their incident links until the degree of all the remaining vertices in the sub-graph are larger than or equal to k. The k layers reflect the connectivity of the ceRNA partners such that the first peeled layer (k=1) contains ceRNAs with only a few connections whereas the last peeled layer is most connected. Each node was assigned to a maximal k core sub-network in each breast cancer subtype ceRNA network. For among different breast cancer subtypes, the layers of each ceRNA network were normalized to 0-1 divided by the number of largest k layer in each network.

### Functional enrichment analysis for subtype common and specific ceRNA hub sub-networks

To dissect the biological pathways represented by the common or subtype-specific ceRNA hub sub-networks, functional enrichment analysis of the hub ceRNA genes was performed by hypergeometric distribution with the Bonferroni correction (FDR < 0.05).

## SUPPLEMENTARY NOTES FIGURES AND TABLES






