# Supplementary material for: Competing endogenous RNA network analysis identifies critical genes among the different breast cancer subtypes
Source: Oncotarget. 2016 Dec 29;8(6):10171–84. doi: 10.18632/oncotarget.14361 (PMC5354650; doi:10.18632/oncotarget.14361)
Supplement: Supplementary file 1 [file oncotarget-08-10171-s001.pdf]

Competing endogenous RNA network analysis identifies critical genes among the different breast cancer subtypes

SUPPLEMENTARY NOTES

Validated expression data for breast cancer subtypes

To validate the co-expression of our identified ceRNA interactions, we obtained another array datasets from the Affymetrix human tissue panel study (including 29 luminal A, 30 luminal B, 30 HER2-enriched and 41 basal-like) which are stored in Gene Expression Omnibus (GEO, GSE45827). After RMA normalization by affy package in R, the probe sets which are annotated to more than two genes were removed. The same genes with more than two probe set values and the replicated samples were averaged together. We calculated the Pearson correlation coefficients for ceRNA interaction pairs identified in each breast cancer subtype. Then the permutation was performed to choose gene pairs randomly, and the number of randomly chosen pairs was identical to the original number of ceRNA interactions. The permuted gene pairs were also tested for Pearson correlation coefficient. This process was repeated 100 times and compared the results with the actual data.

Known tumor suppressive genes (TSGs) and oncogenic genes (OGs)

The known TSGs and OGs were obtained from a previous study, which integrates TCGA mRNA, CNV and mutation data and generated a continuous ranked list for every gene, ranging from more negative (TSGs) to more positive (OGs) with consistent changes across tumors. In our current study, we restricted different score thresholds to get TSGs and OGs for further analysis.

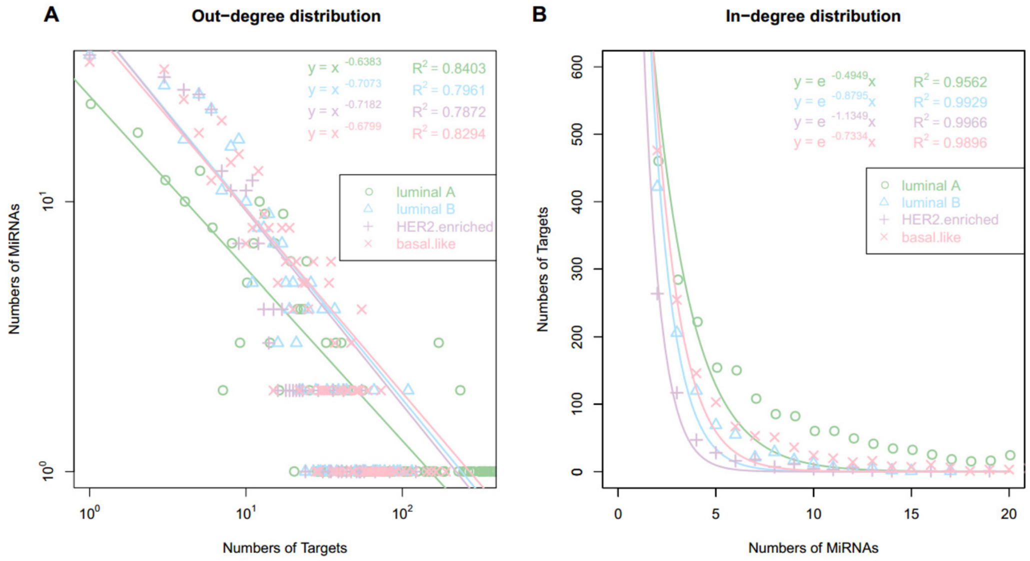

Supplementary Figure 1: Degree distributions of the miRNA dys-regulatory networks. A. Out-degree distributions of the miRNA dys-regulatory networks across five BRCA subtypes. **B.** In-degree distributions of the miRNA dys-regulatory networks across five BRCA subtypes.

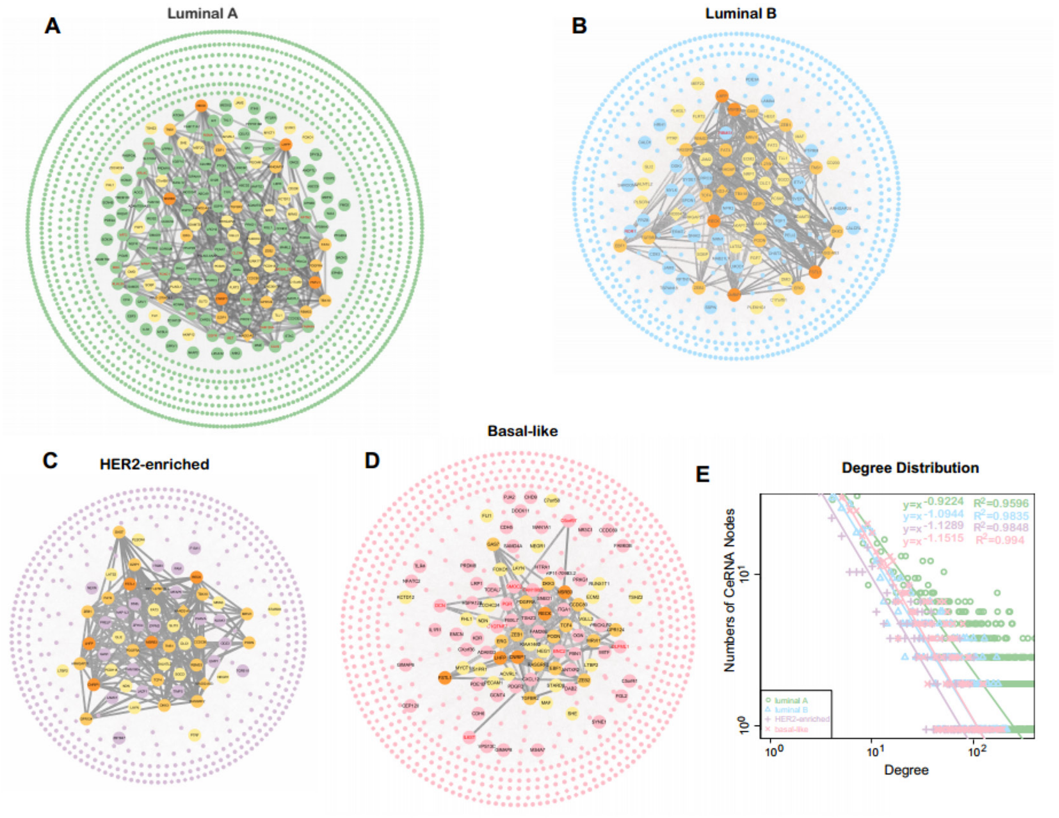

Supplementary Figure 2: CeRNA networks across four breast cancer subtypes. A-D. The maximum components of the whole ceRNA networks across four breast cancer subtypes. Nodes represent ceRNA transcripts, including mRNAs (ellipse) and lncRNAs (diamond). Edges represent positive correlation between ceRNAs. Nodes which are zoomed in represent ceRNA hubs in each subtype. The hubs colored in yellow to orange-red referred to subtype shared ceRNA hubs, otherwise, subtype specific hubs. The redder a hub is, more subtypes in which it is defined as a ceRNA hub. The layout of ceRNA nodes was based on k-core, and the largest k-core was in inner circle. The hubs with red gene label correspond to the 29 critical subtype-specific ceRNA hubs identified later. The interactions among common hubs or interactions among subtype-specific ceRNA hubs were shown in boldface. **E.** Degree distributions of the ceRNA networks across four breast cancer subtypes.

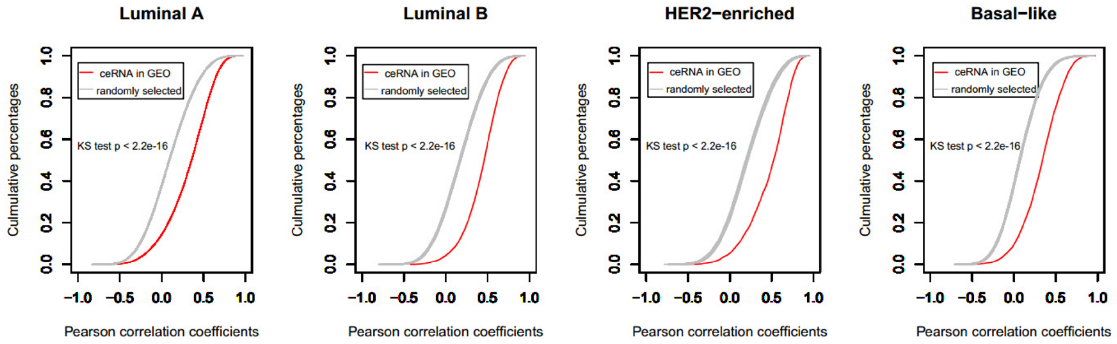

Supplementary Figure 3: Culmulative distribution of the Pearson correlation coefficients for the expression of ceRNA pairs. CeRNA pairs in GEO data (line in red) and gene pairs selected randomly in GEO data (line in grey, 100 lines in grey for 100 repeat times). The KS test was used to calculate a p value for each permutation in each subtype.

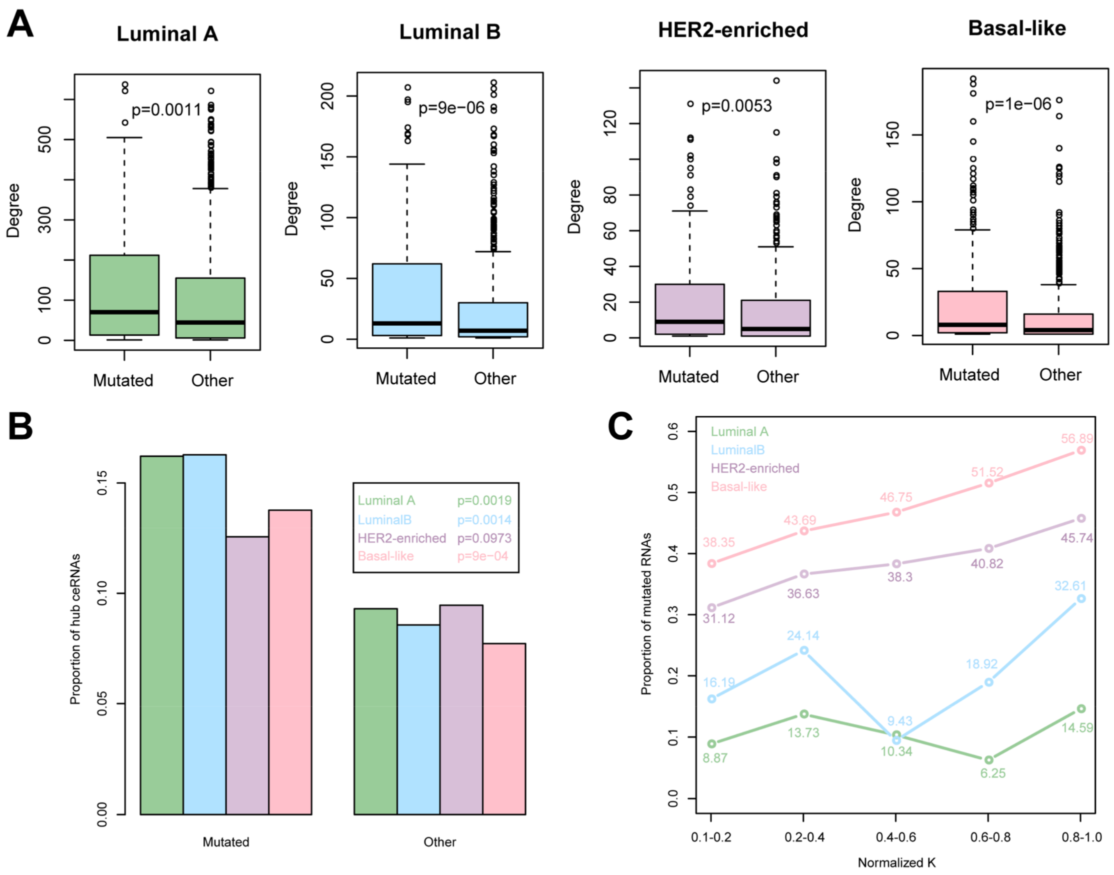

Supplementary Figure 4: Mutated ceRNAs are enriched in ceRNA hubs. A. The difference in degrees between mutated ceRNAs and non-mutated ceRNAs. P-values were calculated by using the Wilcoxon Rank Sum test. **B.** Subtype mutated ceRNAs were enriched in the hubs. Left and right bars respectively represent the proportions of hubs among mutated and non-mutated ceRNAs. P-values were calculated by hypergeometric distribution. **C.** Mutated ceRNAs tend to be in the core of ceRNA networks. Relationships between ceRNA layers and frequency of mutated ceRNAs in each normalized k layer interval. The ceRNAs were classified into five groups by normalized k value, and the percentage calculated by the number of mutated ceRNAs in each group divided by the total ceRNAs in that group.

Supplementary Table 1: The number of clinical samples with RNA expression profiles for four breast cancer subtypes and normal breast tissues

Samples	Luminal A	Luminal B	HER2-enriched	Basal-like	Normal
mRNA	405	187	66	137	104
miRNA	297	135	48	99	83
lncRNA	417	191	67	139	105

Supplementary Table 2: The number of subtype specific dys-regulated RNAs (up/down)

	Luminal A	Luminal B	HER2-enriched	Basal-like
mRNA	132/122	171/266	279/317	985/545
miRNA	1/12	15/7	16/21	76/5
lncRNA	92/208	158/212	136/403	541/444

Note: up refers to up-regulation; down refers to down-regulation.

Supplementary Table 3: The number of nodes and edges of the miRNA dys-regulatory networks across four breast cancer subtypes

	Edges	miRNA	lncRNA	mRNA
Luminal A	14,664	266	738	2,251
Luminal B	4,666	351	572	1,545
HER2-enriched	2,745	294	363	1,067
Basal-like	7,185	392	676	1,766

Supplementary Table 4: The interactions of common ceRNA hub sub-network and the enriched gene sets for common hub sub-network

See Supplementary File 1

Supplementary Table 5: The enriched gene sets for subtype specific ceRNA hub sub-networks

See Supplementary File 2

Supplementary Table 6: Common and subtype specific ceRNA hubs identified by different Pearson correlation coefficient thresholds for the construction of miRNA dys-regulatory networks

	PCC≤0	PCC≤-0.1	PCC≤-0.15	PCC≤-0.2
Luminal A	111	111(111)	111(110)	76(76)
Luminal B	32	32(32)	33(32)	34(32)
HER2-enriched	21	21(21)	21(21)	21(21)
Basal-like	56	56(56)	56(56)	60(56)
Specific(total)	220	220(220)	221(219)	191(185)
Common	28	28(28)	27(27)	27(27)

Note: For the threshold of PCC≤-0.1, -0.15, -0.2, the second number enclosed in parenthesis is the number of common or subtype-specific ceRNA hubs which are also identified by our original thereshold.

Supplementary Table 7: Subtype common and specific ceRNA hub sub-networks are enriched in tumor suppressive genes(TSGs) and depleted in oncogenic genes(OGs)

	OG(≥1) 8601	OG(≥2) 6557	TSG(≤-1) 7235	TSG(≤-2) 4872	TSG(≤-3) 3190	TSG(≤-4) 1903	TSG(≤-5) 1095
Common	-- (1)	-- (1)	1.01e-06 (21)	6.66e-08 (19)	6.99e-08 (16)	7.59e-07 (12)	3.42e-05 (8)
Luminal A	1.00 (13)	1.00 (6)	2.25e-19 (85)	1.05e-16 (67)	9.91e-16 (53)	9.37e-19 (45)	5.98e-10 (25)
Luminal B	0.99 (4)	-- (1)	1.78e-07 (26)	1.18e-07 (22)	1.61e-04 (14)	4.97e-04 (10)	2.50e-01 (3)
HER2-enriched	0.99 (3)	-- (2)	1.32e-02 (13)	4.75e-03 (11)	1.13e-02 (8)	4.18e-02 (5)	2.45e-02 (4)
Basal-like	1 (4)	-- (2)	3.69e-13 (46)	4.31e-15 (41)	1.06e-13 (33)	4.22e-13 (26)	2.55e-09 (17)

Note: Different columns indicated different scores to define OG or TSG sets. The number in the first line means how many TSGs/OGs were defined with different score thresholds. The statistical significances were calculated by hypergeometric tests. The first number represents the enriched p_value ; the second number enclosed in parenthesis is the number of ceRNA hubs which are also in the OG or TSG sets. -- means ignored p values when the number of intersected genes is less than 3.

Supplementary Table 8: TAM analysis for subtype common and subtype-specific miRNA hubs

Subtypes	Category of Gene sets	Term	Count	Bonferroni Corrected_P
Common	Family	mir-8 family	3	2.89e-02
	Function	Human embryonic stem cell (hESC) regulation	8	4.62e-02
	HMDD	Carcinoma of Renal cell	3	4.09e-03
Luminal A	Family	mir-8 family	5	1.10e-05
	Cluster	hsa-mir-182 cluster	3	6.34e-03
	HMDD	Carcinoma of Renal cell	4	8.39e-04
Luminal B	Family	mir-130 family	3	3.86e-02
		mir-15 family	3	3.86e-02
	HMDD	Lymphoma	2	2.48e-03
		Carcinoma of Renal Cell	3	1.08e-02
		Leukemia of B cell	3	1.44e-02
HER2-enriched	Family	mir-17 family	5	1.85e-04
	Function	immune system(Xiao's Cell2009)	6	5.98e-04
		Akt pathway	5	1.21e-02
	HMDD	Neoplasms	10	7.13e-05
		Breast Neoplasms	10	1.87e-03
Basal-like	Family	mir-17 family	6	9.73e-05
	Cluster	hsa-mir-17 cluster	6	3.79e-06
	Function	cell proliferation(Hwang etal BJC2006)	6	3.79e-06
	HMDD	Neoplasms	7	1.20e-04
